# Predicting Bone Metastasis in Prostate Cancer Patients Using Total Serum Prostate-Specific Antigen and Serum Alkaline Phosphatase: Model Development, Validation, and Deployment as a Digital Risk Estimator

**DOI:** 10.7759/cureus.87786

**Published:** 2025-07-12

**Authors:** Frank Obeng, Alfred Yawson, Nii-Boye Hammond, Sylvester A Boakye, Banabas Kpankyaano, Appiateng W Boadu, Evans K Zikpi, Justice Dzomeku, Dominic A Dankwah, James E Mensah

**Affiliations:** 1 Department of Surgery, University of Health and Allied Sciences, Ho, GHA; 2 Department of Community Health, School of Public Health, University of Ghana Medical School, Accra, GHA; 3 Nuclear Medicine, Korle Bu Teaching Hospital, Accra, GHA; 4 Department of Surgery, School of Medicine, University of Health and Allied Sciences, Ho, GHA; 5 Ho Polyclinic, Ghana Health Service, Ho, GHA; 6 Library and Archival Services, University of Health and Allied Sciences, Ho, GHA; 7 Department of Surgery, University of Ghana Medical School, Accra, GHA

**Keywords:** alkaline phosphatase, bone metastasis, clinical decision support tools, digital risk-estimator of bone metastasis in prostate cancer, dre stage, gleason score (isup grade), logistic regression model, prostate-specific antigen, technetium-99 bone scan

## Abstract

Introduction

This study addresses the global controversy over routine bone scans for newly diagnosed prostate cancer patients, focusing on the Ghanaian population. It aims to assess the predictive value of prostate-specific antigen (PSA) for bone metastasis and the role of serum alkaline phosphatase (ALP) in enhancing prediction.

Methods

This study was conducted at Korle Bu Teaching Hospital over 14 months and included 258 treatment-naïve prostate cancer patients. Clinical evaluation, PSA and ALP tests, and technetium-99 bone scans were performed. Chi-square and t-tests identified significant predictors of bone metastasis. The predictors were regressed logistically into a model, which was validated, trained, updated, and programmed into a digital risk calculator. All analysis was at a 0.05 significance level.

Findings

The mean age of participants was 68.18 ± 7.34 years (68.83 and 67.77 years for the metastatic and non-metastatic groups, respectively). Increasing PSA (OR=4.59, p<0.001), ALP (OR=4.24, p<0.001), DRE risk group (OR=1.60, p<0.05), and International Society of Urological Pathology (ISUP) (OR=1.72, p<0.05) were associated with increasing risk of bone metastasis. A two-unit rise along the D-Amico risk strata was associated with a 28-fold increase in the odds of metastasis (low risk vs high risk, OR=29.56, p<0.001; low risk vs intermediate risk, OR=2.80, p=0.368). Among participants with more than 30% of their core-biopsy volume involved with adenocarcinoma, 54.0% had bone metastasis (OR=1.33, p=0.06). Also, 57% of those with perineural invasion had bone metastasis (OR=4.0, p=0.01). Perivascular invasion was not a statistically significant predictor (p=0.346). All patients with cribriform pattern histology had bone metastasis (100%; OR=9.40, p=0.04). Of those with bone pain, 48.4% had bone metastasis (OR=2.25, p=0.002). PSA and ALP exhibited strong independent associations with bone metastasis, with PSA outperforming other predictors (AUC under ROC curve of 81.65% vs 77.97% for ALP, 69.73% for DRE, and 79.19% for ISUP. On multivariate logistic regression, the combined AUC for PSA and ALP was 89.28%, while that for combined PSA, ALP, DRE, and ISUP was 92.3%). A PSA cut-off of 18.95 ng/mL or an ALP cutoff of 59.48 IU/L, individually, detected 97.5% of bone metastasis. Combining a PSA cut-off of 20.85 ng/mL with an ALP cut-off of 44.0 IU/L yielded a 100% detection rate, while PSA above 20 ng/mL, DRE >T2c, and Gleason score >7 predicted 95% of bone metastasis in this cohort. In a logistic regression model, ALP, ISUP, and DRE stage significantly improved the bone metastasis detection rate of PSA in prostate cancer (z-statistic gain at p=0.05 was 2.41; new AUC=92.29%). The inclusion of bone pain, cribriform pattern histology, perineural invasion, and percentage core involvement of adenocarcinoma yielded just marginal gains (maximum z-statistic gain at p=0.05 was 0.61; new AUC: 93.90%).

Conclusion

For high-risk prostate cancer, bone scans are recommended. In Ghana, a PSA cut-off of 18.95 ng/mL could safely exclude 20% of bone scans, missing only 2.5% of bone metastases, saving $170 per patient ($55,000 nationally per year). Combining this with an ALP cut-off of 44.0 IU/L (0% false-negative rate) detects 100%. A validated risk model combining PSA, ALP, ISUP, and DRE achieves an AUC of 92.3%, sensitivity of 89.9%, specificity of 78.8%, and accuracy of 85.6%, offering a practical, digitally deployed, decision support tool.

## Introduction

Prostate cancer is the most frequently diagnosed non-cutaneous malignancy among men in many Western countries [1-5]. In Ghana, it is the second most common cancer in men, responsible for 7% of all male cancers and 17.35% of male cancer-related deaths [4,6,7]. At Korle Bu Teaching Hospital, Accra, Ghana, prostate cancer is the predominant genitourinary malignancy, comprising nearly two-thirds of such tumors from 1980 to 1990 [6].

Prostate cancer diagnosis and staging involve taking a comprehensive history, performing a digital rectal examination (DRE), measuring serum prostate-specific antigen (PSA), performing transrectal ultrasound (TRUS)-guided prostate biopsy, and performing histological grading using Gleason or International Society of Urological Pathology (ISUP) score [1-22]. For staging purposes, technetium-99m methylene diphosphonate (99mTc-MDP) bone scintigraphy remains the gold standard for detecting bone metastasis, which is the most common site of spread in advanced disease. Bone metastasis in prostate cancer is usually osteoblastic and may make assays of serum alkaline phosphatase (ALP) useful [8-19,21-27].

Globally, the routine use of bone scans for all newly diagnosed prostate cancer patients is debated [12-16]. The European Association of Urology (EAU) and the American Urological Association (AUA) differ only slightly in their recommendations [12], and various studies from Asia and Africa have proposed different PSA cut-off values for bone scan indications [8,11-13,15,16]. Both the AUA (2023) and EAU (2023) guidelines recommend limiting bone scan investigations to prostate cancer patients with higher risk profiles. Specifically, the AUA advises bone scans for patients with PSA >20 ng/mL, Gleason scores ≥8/grade group 4-5, or clinical stage ≥T3a [3, 12]. Similarly, the EAU does not recommend bone scans for asymptomatic patients with well or moderately differentiated tumors if PSA is below 20 ng/mL. These thresholds support targeted imaging based on PSA level, Gleason grade, and clinical stage [3,12].

For intermediate-risk patients - those with PSA between 10-20 ng/mL, Gleason scores of 7 (3+4 or 4+3), or clinical stage T2b-T2c - a bone scan should be considered if symptoms of bone pain exist [3,12]. Among patients on active surveillance, a rising PSA or new clinical concerns may prompt a bone scan, though this should be based on clinical judgment. In low-risk individuals - defined by PSA levels below 10 ng/mL, Gleason scores ≤6, and localized clinical staging - a bone scan is not typically recommended, especially in the absence of symptoms [12]. Likewise, asymptomatic patients with no high - or intermediate-risk features do not require routine bone imaging [12]. Suffice it to say, however, that in all patients earmarked for radical prostatectomy or other treatment modalities with curative intent, a staging bone scan should be considered.

In Ghana, this discussion is even more critical due to infrastructural and economic constraints. The cost of a bone scan is approximately GHS 1,800 (170 USD), a burden for many patients who already bear out-of-pocket costs for TRUS biopsy and other staging tests. Additionally, the bone scan procedure involves injection of a radiotracer, a 3-hour waiting period, and 30 minutes of scanning, all of which can be physically and psychologically taxing, especially for frail patients [28].

Despite the EAU guideline PSA threshold of 20 ng/mL [12], studies show significant variability in PSA performance across different populations [12,15,19]. This necessitates context-specific evidence. Furthermore, serum ALP - a marker of osteoblastic activity - may enhance PSA’s predictive performance, though African data are sparse [23,24].

Multiple studies across diverse populations have investigated the predictive value of PSA and ALP in determining bone metastasis in newly diagnosed prostate cancer patients [8-28]. Al-Ghazo et al., in a cohort of 106 patients, reported that those with PSA ≤20 ng/mL and Gleason scores <8 typically had negative bone scans, suggesting that both PSA and Gleason score are reliable predictors of metastatic spread, though the retrospective design limits causal inference [8]. Similarly, a study by Megumi Hirobe et al. involving 366 patients found no metastasis in those with PSA <10 ng/mL and only 0.5% metastasis in those with PSA between 10 and 20 ng/mL, but the overall metastasis rate was low (7.7%), potentially limiting generalizability due to small event numbers [26].

In a Taiwanese study by Wei et al., PSA levels below 13 ng/mL correlated with a very low likelihood of metastasis, with the model achieving 96.43% sensitivity and 84.09% specificity; however, the authors did not establish a definitive cut-off [22].

In Africa, Ojuka et al. studied 54 Kenyan patients and found a 20% rate of positive bone scans, with a significant correlation to Gleason scores (p < 0.001), though the small sample size and undefined PSA cut-off values limited the precision of their conclusions [13]. Hammond et al. conducted one of the largest West African studies with 363 patients, demonstrating that PSA and Gleason score were significant predictors of bone metastasis. They proposed a PSA cut-off of 20 ng/mL, achieving 86.5% sensitivity and 41.2% specificity, although some metastatic cases were missed at lower PSA levels [27].

Finally, Kamaleshwaran et al. in a study of 322 patients found that 70% of patients with PSA >100 ng/mL had positive bone scans, while 97.4% of those with PSA <20 ng/mL showed no metastasis. However, this retrospective design was susceptible to selection bias, highlighting the need for prospective validation [16]. Collectively, these studies reinforce PSA’s role as a primary predictor while supporting the supplementary utility of ALP, particularly in resource-limited contexts.

From the foregoing, African studies from Ghana and Kenya (e.g., Hammond et al. and Ojuka et al.) have explored PSA's utility but are limited by retrospective design or small sample sizes [13,27]. Ghanaian studies exploring the possible role of ALP are also needed. There is also a need for locally validated models that incorporate PSA, ALP, ISUP, and DRE to guide bone scan decisions.

The aim of this study was to determine the usefulness of total serum PSA and serum ALP in predicting the risk of bone metastasis among newly diagnosed and treatment-naïve prostate cancer patients at Korle Bu Teaching Hospital.

The objectives of the study were to determine and categorize total serum PSA of treatment-naïve prostate cancer patients and correlate the PSA categories (0-4, 4.1-10, 10.1-20, 20.1-100, >100 ng/mL) with bone scan results. We also correlated total serum ALP, DRE clinical stage, and Gleason Score individually with bone scan outcomes. The other objectives were to evaluate whether combining PSA, ALP, DRE, and Gleason Score, with the additional parameters, cribriform pattern, perineural/perivascular invasion, percentage core involvement, and the symptom of bone pain improves the predictive accuracy for bone metastasis beyond PSA alone and to design a predictive model (risk estimator) out of it. The final objective was to identify PSA and ALP categories that can define a subgroup of newly diagnosed patients in whom we can safely omit a bone scan investigation.

Hypothesis

H1: Increasing total serum PSA is associated with a higher likelihood of bone metastasis.

H2: Elevated serum ALP is associated with an increased risk of bone metastasis.

H3: The combined predictive value of ALP and PSA is superior to PSA alone.

H4: Higher DRE clinical stage and ISUP grade correlate with increased bone metastasis risk.

H5: The combined predictive value of DRE, ISUP, serum ALP, and serum PSA is greater than that of serum PSA and ALP (together) in determining bone metastasis.

H6: Combining serum PSA, ALP, DRE, ISUP, bone pain, a histology of cribriform pattern, perineural/perivascular invasion, and the percentage core involvement of adenocarcinoma (eight-parameter model) yields a statically significant improvement in determining bone metastasis in prostate cancer than the combined serum PSA, ALP, ISUP, and DRE (four-parameter model).

Previous presentation

Preliminary findings from this study were presented at the 2024 Annual Scientific Conference of the University of Health and Allied Sciences, Ho, Ghana, where feedback from experts guided the final analytic model and digital risk estimator design.

It is also part of a larger Fellowship Degree Thesis (as a partial requirement for the conferment of "Fellowship of the Ghana College of Physicians and Surgeons" [Urology]), and available in Preprint [28].

## Materials and methods

Study design and participants

This was a hospital-based cross-sectional study with a longitudinal follow-up period of three months, conducted among newly diagnosed, treatment-naïve prostate cancer patients. The study design allowed prospective data collection on diagnostic parameters, laboratory values, and bone scan outcomes, enabling the evaluation of predictors for bone metastasis at baseline and early follow-up.

Study setting and population

Setting

The study was conducted at the Korle Bu Teaching Hospital, specifically at the Urology Unit of the Department of Surgery and the Nuclear Medicine Unit of the Radiology Department. Patients were enrolled as they presented to the urology unit for clinical evaluation leading to the need for a bone scan examination. At the nuclear medicine unit, other patients referred from other hospitals were recruited as they presented for booking for bone scan examination.

Participants

The study population included adult male patients newly diagnosed with histologically confirmed prostate cancer presenting to Korle Bu Teaching Hospital between January and December 2018. All participants were treatment-naïve at the time of inclusion.

Variables

Key variables extracted included demographics, such as age and address, and clinicopathological parameters, such as PSA level at diagnosis (ng/mL), serum total ALP (IU/L), DRE findings and clinical T-stage, Gleason score and ISUP grade from TRUS-guided biopsy, together with cribriform pattern, perineural/perivascular invasion, percentage core involvement, and the symptom of bone pain. The presence or absence of bone metastasis on 99mTc-MDP bone scan and metastatic pattern (focal vs. diffuse) was also considered.

Eligibility criteria

Inclusion

The study included male patients aged ≥40 years, with histologically confirmed adenocarcinoma of the prostate, who were treatment-naïve and undergoing a staging bone scan investigation. Participants also provided consent for PSA and ALP tests before inclusion in the study.

Exclusion

Patients already on androgen deprivation therapy or radiotherapy, patients with incomplete diagnostic or laboratory data and non-consenting individuals, and patients with deranged liver function tests suggesting hepatobiliary disease, notably alanine aminotransferase, aspartate aminotransferase, and gamma-glutamyl transferase, were excluded.

Data sources/measurement

Patient records, laboratory databases, and imaging reports were reviewed. PSA and ALP values were determined from venous blood samples using standard automated assays. Bone scans were interpreted by two independent nuclear medicine specialists blinded to clinical data. In analyzing hotspots and interpreting 99mTc-MDP bone scans for prostate cancer, maintaining diagnostic sensitivity and specificity, we used a structured, stepwise quality control (QC) protocol usual to nuclear physicians. It covers radiopharmacy, imaging, analysis, and reporting. The process begins with verifying radiotracer integrity via thin-layer chromatography to confirm ≥95% labeling efficiency, confirming sterility, and administering an appropriate dose (740-925 MBq), with complete documentation of dose, timing, and residual. Patients are hydrated and asked to void, and bone-affecting agents (e.g., bisphosphonates) are noted or paused.

Gamma camera QC includes daily uniformity checks (≤5%), weekly energy calibration (140 keV ±2%), monthly resolution tests, and quarterly CT-to-SPECT attenuation alignment (±5 HU). Acquisition involves planar scans at 10-12 cm/min (256 × 1024 matrix), motion minimization (<1 pixel drift), and optional marker placement. SPECT/CT is used where indicated, reconstructed using standard Ordered Subset Expectation Maximization (OSEM) algorithms, with image integrity verified via Digital Imaging and Communications in Medical reporting (DICOM) headers and real-time technologist review. Bed alignment, co-registration accuracy, and metadata completeness are confirmed before images are transferred to PACS (Picture Archiving and Communication System).

The nuclear physicians begin by reviewing planar images for distribution patterns - metastasis often appears as asymmetric, multifocal axial uptake, while benign changes are periarticular and symmetric. SPECT/CT, when available, adds anatomical localization, while lesion-to-normal (L/N) count ratios (>3-4) aid in interpretation. Correlation with prior imaging, PSA, ALP, and histologic grade strengthens diagnostic confidence. Reporting uses structured templates to classify each lesion as malignant, benign, or equivocal, with difficult cases reviewed by two other peers or in multidisciplinary settings. When hotspots are equivocal, tie-breakers follow a hierarchy: first, targeted SPECT/CT; then, focused radiographs or CT; followed by MRI with diffusion-weighted imaging (DWI) if marrow detail is needed. PSMA-PET/CT or 18F-NaF PET may be used when available (such patients may be referred to other centers that have it for the purpose). Stable lesions may be re-imaged after three to six months; biopsy is reserved for critical single lesions. A lesion is termed indeterminate only when no further clarification is feasible, with clear follow-up plans documented.

Post-report QC includes periodic audits, discrepancy reviews, and sensitivity-specificity tracking (targets: ≥90% sensitivity, ≥80% specificity). Structured DICOM-SR exports support AI applications and standardization. Through consistent QC from radiotracer preparation to final reporting and audit, centers like ours ensure that hot spots called metastatic are truly so and those called benign are reliably excluded, thereby preserving diagnostic performance in clinical decision-making.

Bias

A uniform data extraction template was used. Two independent reviewers performed double entry and cross-checking to ensure accuracy and reduce selection and information bias.

Data collection tool

A standardized questionnaire was designed to capture demographic, clinicopathological, laboratory, and imaging data in a consistent format across all participants.

Data collection

Data were collected prospectively by the lead author and trained research assistants from urology and nuclear medicine records. Each patient was followed up for up to three months to assess any delayed bone scan reporting or interim serum marker changes.

Sample size and sampling

Using Cochran’s formula and based on a prior prevalence of 20% for bone metastasis among newly diagnosed prostate cancer patients [17], a minimum sample size of 246 was calculated. Adjusting for 5% non-response, a total of 258 participants were targeted. Consecutive sampling was used until the sample size was achieved.

Data analysis/statistical methods

Descriptive statistics (means, medians, frequencies, proportions, percentages) were used to summarize demographic and clinical data. Bivariate analyses (chi-square, t-test) assessed associations between PSA, ALP, DRE, ISUP, and bone metastasis. Multivariate logistic regression identified independent predictors.

Multivariate regression analyses used backward stepwise selection (p < 0.05 inclusion). Diagnostic performance was assessed using sensitivity, specificity, positive predictive value (PPV), negative predictive value (NPV), and area under the ROC curve (AUC). Analyses were conducted using Stata Version 17 (StataCorp LLC, College Station, TX), with a significance level set at p < 0.05.

Technical information

Model Development

Logistic regression analysis was used to develop the prediction model using significant variables (p < 0.05). Model performance metrics including AUC, Hosmer-Lemeshow test, and calibration plots were computed. Internal validation (70% of the data) and model training (10% of the data) were performed. External validation was conducted using bootstrapping and a reserved 30% of the dataset.

Mobile Application Development

The validated model was programmed into a mobile app using R Shiny, which stratifies patients into low- or high-risk categories and gives tailored risk outputs for bone metastasis. The app was deployed for Web and Android and is usable offline.

Pilot Testing

The app was piloted with 15 users (15 urologists/general practitioners).

Ethical approval

Ethical clearance for the research was obtained from the Korle Bu Teaching Hospital Institutional Review Board and Ethical Research Committee. The approval number was KBTH-IRB/00086/2018 (Korle Bu Teaching Hospital). All procedures involving human participants were conducted in accordance with the ethical standards of the institutional and/or national research committee and the 1964 Helsinki Declaration and its later amendments or comparable ethical standards. Written informed consent was obtained from all participants prior to their inclusion in the study. Throughout the study, strict measures were taken to ensure patient anonymity and confidentiality, including the use of de-identified data and secure data handling protocols.

## Results

Sociodemographic characteristics/analysis

**Figure 1:**
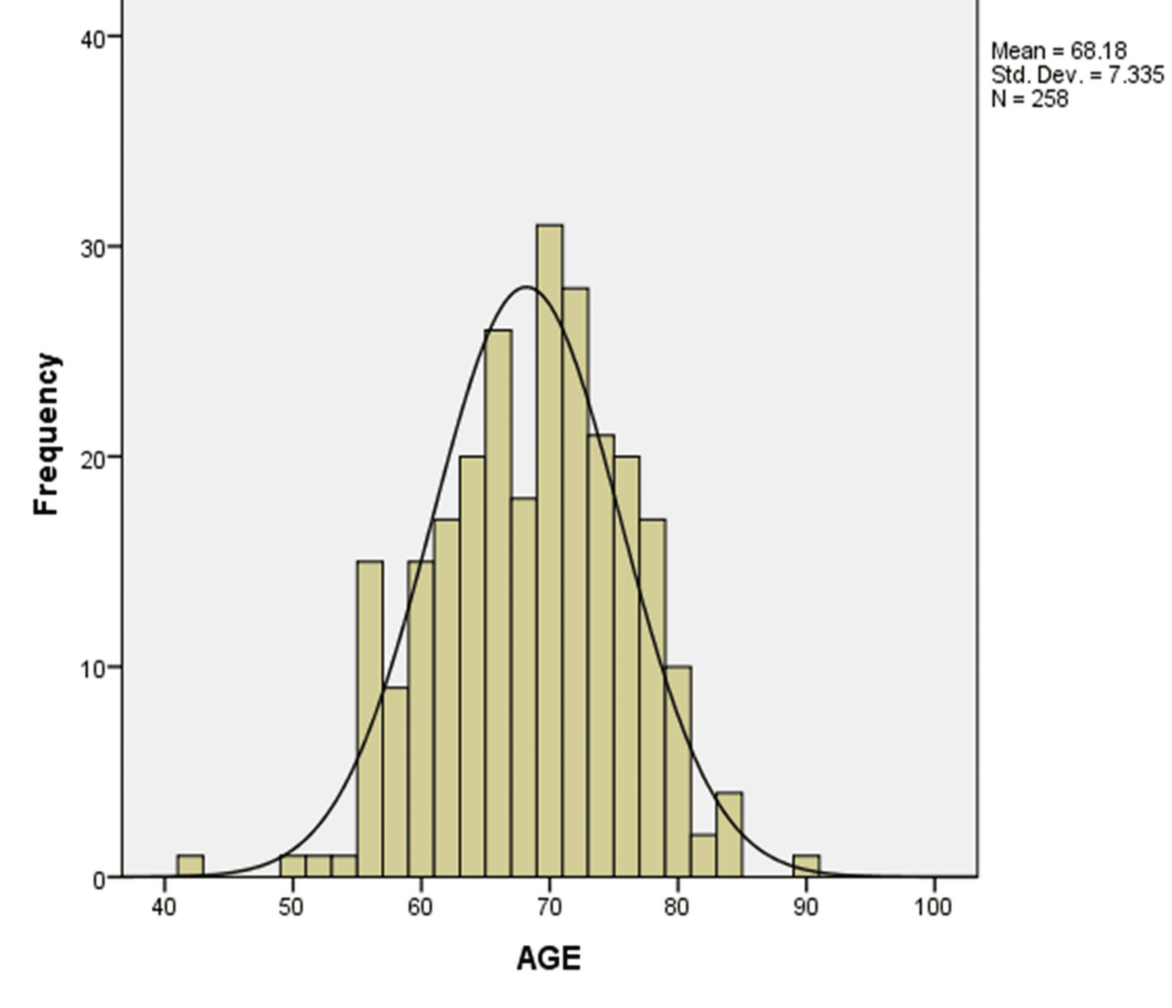
Age distribution of prostate cancer patients included in the study (N = 258) This histogram illustrates the distribution of patient ages in the population of newly diagnosed prostate cancer patients, accessing technetium-99 bone scan service at Korle Bu Teaching Hospital. The data show a near-normal distribution, with a slight right skew. Superimposed on the histogram is a normal distribution curve for visual comparison. The mean age of participants was 68.18 years, with a standard deviation of 7.34 years, and age ranged from approximately 42 to 95 years. The highest frequency of patients fell within the age bracket of 65–75 years, consistent with the known epidemiology of prostate cancer as a disease predominantly affecting older men. This distribution supports the representativeness of the sample in capturing the age profile of patients at risk for advanced prostate cancer and bone metastasis.

For the comparative aspects of the descriptive analysis in this study, the metastatic group was considered the study group, while the non-metastatic group was considered the control group. The age distribution of all participants in this study approximated a normal curve (Figure 1). The mean age was 68.18 years, which nearly coincided with the median age of 69 years; the modal age was 65 years, and the age range was 47 (45 to 92) years. In this study, patients with bone metastasis constituted the study group, while those without bone metastasis constituted the control group. The study group consisted of 100 individuals, and the control group had 158 individuals (Table 1). This 1:1.6 recruitment ratio reflected the typical pattern at the Nuclear Medicine Unit of Korle Bu Teaching Hospital, where more patients tend to return negative bone scan results than positive ones.

**Table 1:** Summary statistics and independent t-tests for continuous variables This table presents a comparative analysis of the values of PSA), ALP, and age between SG and CG. For each parameter, the number of participants in each group, the mean, the SD, and the SEM are shown. The equality of variances between groups was assessed using Levene’s test, with corresponding F and p-values reported. Independent samples t-tests were conducted to compare means between groups under the assumption of either equal or unequal variances, as determined by Levene’s results. The t-values and df for both assumptions are included for each parameter. PSA, prostate-specific antigen (a biomarker commonly used in prostate cancer assessment); ALP, alkaline phosphatase (an enzyme linked with bone metastasis); SG, study group; CG, control group; SD, standard deviation (indicating variability within each group); SEM, standard error of the mean; df, degrees of freedom associated with the statistical tests

Parameter	Group SG, N	Group SG, Mean ± SD	Group SG, SEM	Group CG, N	Group CG, Mean ± SD	Group CG, SEM	Levene's F	Levene's p	t-Value (Equal Variance)	df (Equal Variance)	t-Value (Unequal Variance)	df (Unequal Variance)
PSA (ng/mL)	100	915.3 ± 2644.55	264.4553	158	54.84 ± 173.57	13.8087	37.505	0.000	4.081	256	3.249	99.54
ALP	100	238.24 ± 436.51	43.6511	158	80.88 ± 25.16	2.0019	43.328	0.000	4.524	256	3.601	99.417
Age	100	68.83 ± 7.04	0.704	158	67.77 ± 7.51	0.597	0.3	0.584	1.136	256	1.152	220.643

The majority of participants had tertiary education (48%), 28% had secondary education, and 13.2% had only basic education. Only 3.2% reported no formal education. Regarding ethnicity, the Akan ethnic group dominated, representing 59% of the cohort. Ga-Adangbe and Ewe ethnicities were also represented, making up 19% and 15%, respectively. Participants from the northern parts of Ghana made up just 7%, and the least represented were the Guans. Alcohol consumption was reported by 52% of participants, while 11.6% had a history of cigarette smoking. Among the smokers, the majority (6.6%) had, cumulatively, smoked less than 10 pack-years.

Descriptive analysis

**Figure 2:**
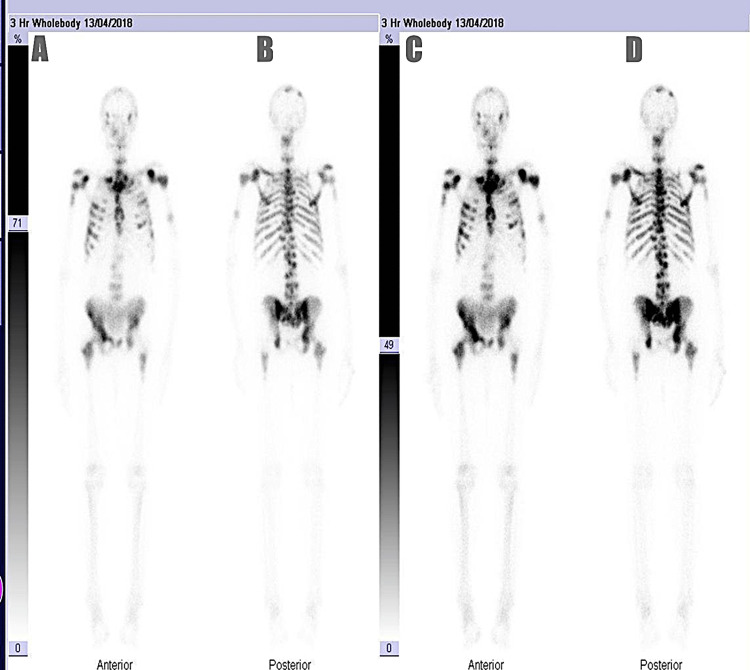
Representative image of a positive 99mTc-MDP whole-body bone scan in a prostate cancer patient with metastatic disease This figure displays the anterior and posterior whole-body bone scan images acquired 3 hours post-intravenous injection of 99mTc-MDP, a radiotracer used in skeletal scintigraphy. The scan was performed on 13/04/2018 as part of the staging evaluation for prostate cancer. The images A and B show initial whole-body anterior and posterior views respectively, while C and D demonstrate enhanced contrast rendering for clearer visualization of skeletal uptake patterns (C for anterior view and D for posterior view). Both sets reveal multiple foci of increased radiotracer uptake, predominantly in the axial skeleton - including the thoracic spine, lumbar spine, ribs, sternum, pelvis - and proximal femora. These areas of increased uptake are highly suggestive of osteoblastic bone metastases, which are characteristic of advanced prostate cancer dissemination to the skeleton. This positive scan confirms metastatic skeletal involvement and underscores the importance of bone scintigraphy in staging and therapeutic planning for patients with elevated PSA, high ISUP grades, or clinical suspicion of metastatic disease. 99mTc-MDP, technetium-99m-labeled methylene diphosphonate; PSA, prostate-specific antigen; ISUP, International Society of Urological Pathology

**Figure 3:**
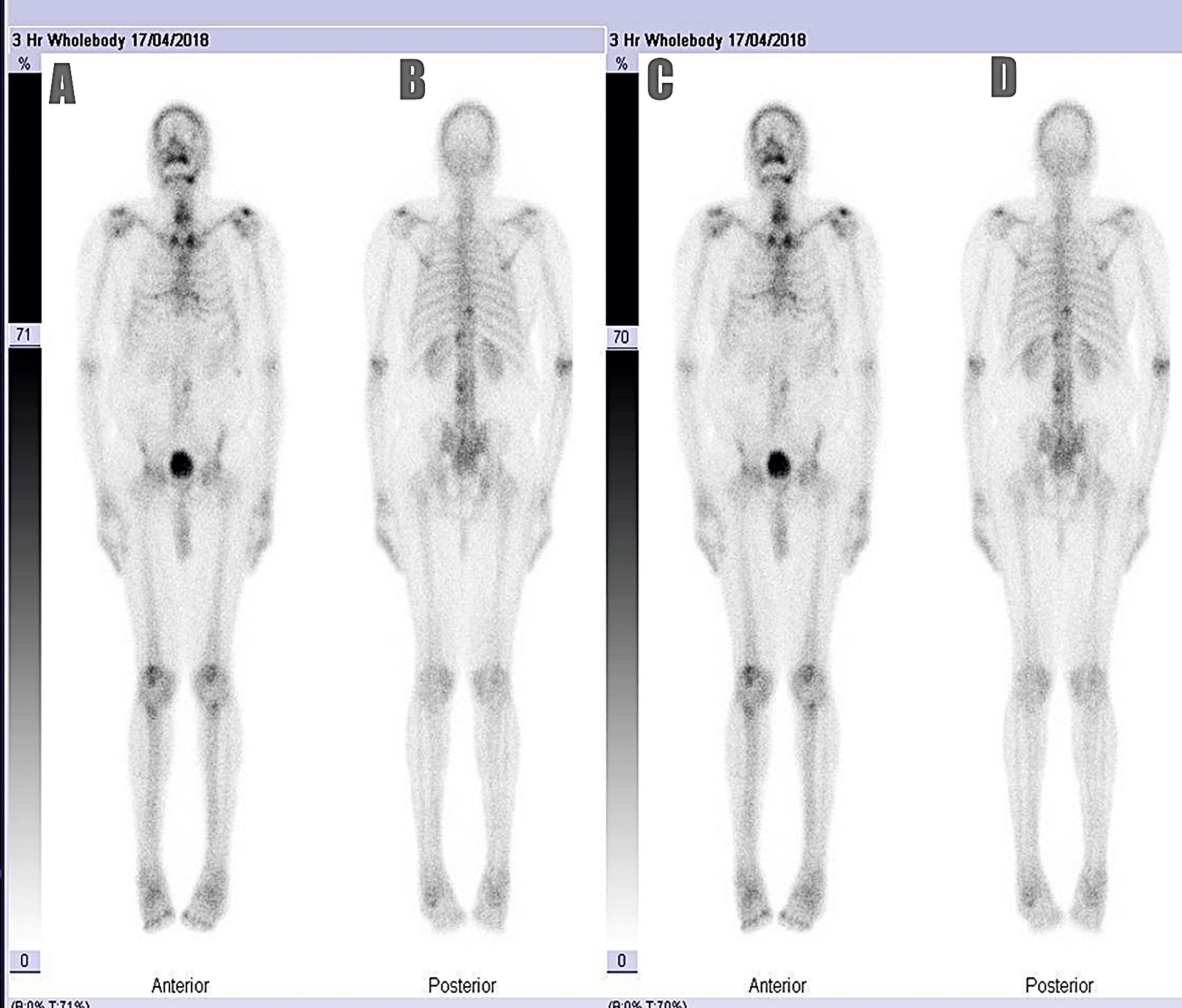
Representative image of a negative 99mTc-MDP whole-body bone scan in a prostate cancer patient without evidence of skeletal metastases This figure displays anterior and posterior whole-body bone scintigraphy images taken 3 hours post-intravenous administration of 99mTc-MDP. The scan was performed on 17/04/2018 as part of the initial metastatic work-up in a prostate cancer patient. The images A and B represent unenhanced anterior and posterior scintigraph views, respectively. The images C and D, on the other hand, represent enhanced anterior and posterior scintigraph views, respectively. The different contrast adjustments are for optimal visualization. The distribution of radiotracer activity demonstrates physiological uptake in areas such as the joints, kidneys, and urinary bladder, without any evidence of abnormal focal uptake suggestive of osteoblastic metastatic lesions. The absence of tracer accumulation in the axial and appendicular skeleton confirms a negative bone scan, indicating no scintigraph evidence of skeletal metastases. Bone scans such as these are instrumental in appropriately staging patients and avoiding unnecessary treatment intensification in the absence of metastatic disease. 99mTc-MDP, technetium-99m-labeled methylene diphosphonate

In total, 38.8% of the study participants had a positive bone scan result indicative of metastasis (Figures 2, 3). Out of the 258 participants, only 13 had prior lumbosacral spine X-rays. Regarding family history, 21.3% of participants reported a positive history of prostate cancer in a male relative, with brother being the most commonly affected relative (8.1%). One participant reported that his son was diagnosed with prostate cancer prior to his own diagnosis. In terms of clinical symptoms, 47.3% of participants had bone pain prior to their bone scan investigation. The distribution of statistics of PSA, ALP, ISUP, and DRE clinical stage among the metastatic and non-metastatic groups is shown in Table 2.

**Table 2:** Descriptive statistics This table summarizes key descriptive statistics for ALP, PSA, ISUP grade, and DRE-based clinical risk among patients with and without bone metastasis. Metrics include minimum, percentiles, quartiles, median, maximum, mode, mean, and standard deviation where applicable. ALP, alkaline phosphatase; PSA, prostate-specific antigen; ISUP, International Society of Urological Pathology grade group; DRE_Risk, disease risk level based on digital rectal examination findings; Mets, metastasis; NA, not applicable; Q1, first quartile; Q3, third quartile

Variable	Group	Min	2.5th Percentile	5th Percentile	Q1	Median	Q3	95th Percentile	97.25th Percentile	Max	Mode	Mean	Std Dev
ALP	Mets absent	40	46.925	50	64	77.5	92	125.2	135.048	222	73	80.7278	24.85
PSA	Mets absent	0.04	4.9512	5.785	13.49	25.21	41.025	146.469	200.27	2076.5	18	54.8613	173.567
ISUP	Mets absent	1	1	1	1	2	3	4	4.7375	5	1	NA	NA
DRE_Risk	Mets absent	1	1	1	1	1	3	3	3	3	1	NA	NA
ALP	Mets present	45	59.475	62.627	83.5	105.5	169.5	1018.65	1437.37	3265	100	238.477	436.452
PSA	Mets present	5.76	18.95	20.855	63.5025	158.5	548.25	4116.5	6021.45	22223	100	915.252	2644.57
ISUP	Mets present	1	1	1	3	4	5	5	5	5	4	NA	NA
DRE_Risk	Mets present	1	1	1	2	3	3	3	3	3	3	NA	NA

Analysis from Table 3 demonstrates that there was no statistically significant association between bone metastasis and factors such as age, family history, smoking, and perivascular invasion of adenocarcinoma on histology for their prostate core biopsy investigation, as all corresponding p-values were above 0.05 (Table 3).

**Table 3:** Chi-square analysis between bone scan results, age, and known pathological parameters related to carcinoma of the prostate PSA, prostate-specific antigen; ISUP, International Society of Urological Pathology grade group; DRE_Risk, disease risk level based on digital rectal examination findings

Parameter Being Tested	Likelihood Ratio	Pearson’s Chi-Square	Degrees of Freedom	Level of Significance
Age	38.516	31.936	34	0.220
Number of pack years of smoking	16.591	12.565	12	0.166
Bone pain	9.088	9.007	2	0.011
PSA	305.33	229.512	199	0.000
%Volume of biopsy cores with adenocarcinoma on histopathology	53.72	40.12	36	0.029
Perineural invasion	7.667	7.505	2	0.029
Perivascular invasion	2.122	1.812	2	0.346
Cribiform/intraductal neoplasia	9.635	8.056	2	0.042
ISUP grade	70.31	66.5	4	0.000
Serum alkaline phosphatase	221.9	168.33	114	0.000
DRE categories	35.747	34.85	2	0.000

Prevalence estimates

The prevalence of bone metastasis among the study cohort was 38.8%, meaning more than a third of newly diagnosed and treatment-naïve prostate cancer patients had already developed skeletal metastases detectable on technetium-99 bone scintigraphy.

Risk factor analysis

On bivariate analysis, independent t-tests (Table 3) showed that the mean ages for the metastasis group (68.83 years) and the non-metastasis group (67.77 years) were not significantly different (p = 0.25). On t-test for difference between group means, patients with skeletal metastases had significantly higher mean PSA levels (915.30±2644.55 ng/mL) compared to those without metastases (58.84±173.57 ng/mL; p = 0.002). First-visit serum ALP was also markedly higher in metastasis-positive patients (238.24±436.51 IU/L) than in those with no metastasis (80.88±25.16 IU/L; p = 0.002). Test of associations showed statistically significant associations between bone metastasis, and total serum PSA, serum ALP, bone pain, cribriform pattern, perineural invasion, percentage of volume of biopsy with tumor involvement, DRE categories, ISUP categories, and D-Amico strata (Table 3).

A two-unit rise along the D-Amico risk strata was associated with a 28-fold increase in the odds of metastasis (low risk vs high risk, OR = 29.56, p < 0.001; low risk vs intermediate risk (OR = 2.80, p = 0.368). Overall, 54.0% of participants with greater than 30% of the volume of their core biopsy involved with adenocarcinoma (OR = 1.33, p = 0.06) had bone metastasis, and 57% of those with perineural invasion (OR = 4.0, p = 0.01) had bone metastasis. Perivascular invasion did not have a statistically significant association with bone metastasis ab initio (p = 0.346). The rare cribriform pattern histology was uniformly associated with bone metastasis (100%) in the small sub-cohort that had it (OR = 9.40, p = 0.04), and 48.4% of those with bone pain (OR = 2.25, p = 0.002) had bone metastasis. There was no significant difference in age between the groups (p = 0.250).

Establishing clinically useful PSA and ALP cut-off points

As shown by the calculated intra-row percentages in Table 4 for "bone metastasis," as predicted by the stated categories of the independent parameters (PSA, DRE, ISUP, D-Amico, bone pain, cribriform pattern, perineural invasion, percentage core involvement, and ALP), as the categories increase in value, the yield for bone metastasis also increases: for serum PSA, 0% for the 0 to 4 ng/mL group to 93% for the >500 ng/mL group. The picture for total serum ALP is also the same, increasing from 0% for the 0 to 32 mm/L group to 100% for the >222 ng/mL group. Therefore, we set out to analyze the 95th and 97.5th percentiles for cut-offs for the two parameters.

**Table 4:** Distribution of bone scan results across PSA and ALP categories and D’Amico risk groups ALP, alkaline phosphatase; PSA, prostate-specific antigen; ISUP, International Society of Urological Pathology grade group; Hist, histology; DRE_Risk, disease risk level based on digital rectal examination findings

Group Type	Category	Metastasis Present (%)	Metastasis Absent (%)	Total (%)
PSA category	0–4	0%	100%	100%
	4.0–10	3.20%	96.80%	100%
	10.1–20	9.80%	90.20%	100%
	20.1–100	34.20%	65.80%	100%
	100.1–500	69.00%	31.00%	100%
	>500	93.10%	6.90%	100%
Bone pain	No	29.40%	70.60%	100%
	Yes	48.40%	51.60%	100%
DRE risk	Low risk	20.20%	79.80%	100%
	Intermediate risk	30.60%	60.40%	100%
	High risk	58.10%	41.90%	100%
ISUP risk	Low risk	12.70%	87.30%	100%
	Intermediate risk	31.60%	68.40%	100%
	High risk	71.40%	28.60%	100%
D'Amico risk	Low risk	3.50%	96.50%	100%
	Intermediate risk	9.10%	90.90%	100%
	High risk	52.80%	47.20%	100%
ALP category	0–32	0%	0%	100%
	32.1–62	11.90%	88.10%	100%
	62.1–92	22.90%	77.10%	100%
	92.1–122	52.50%	47.50%	100%
	122.1–222	70.00%	30.00%	100%
	>222	100%	0%	100%
Hist volume	<30%	34.91%	65.09%	100%
	30–50%	58.33%	41.67%	100%
	>50%	53.12%	46.88%	100%
Perineural invasion	Absent	35.59%	64.41%	100%
	Present	57.14%	42.86%	100%
Cribriform pattern	Pattern absent	37.55%	62.45%	100%
	Pattern present	100%	0%	100%

When the 2.5th and the 5th percentiles were analyzed for cut-off points for total serum PSA and ALP for which a technetium bone scan investigation can be omitted (Table 2), the following results were obtained: 18.95 ng/mL and 59.48 IU/L, respectively, at the 2.5th percentile. Values of 20.85 ng/mL and 62.03 IU/L were obtained at the 5th percentile for total serum PSA and total serum ALP, respectively (Table 2). The lowest ALP level at which there was metastasis in the cohort was 45.0 IU/L, while the lowest PSA at which there was bone metastasis was 5.76 ng/mL (Table 2).

In our cohort, a PSA cut-off of 10.0 ng/mL would correctly identify 99.0% of all bone metastasis (Table 4). Combining this with an ALP cut-off of 44.0 IU/L could lead to a correct identification of all metastatic cases (100.0%). On the other hand, a PSA cut-off of 18.95 ng/mL would correctly identify 97.5% of all bone metastasis, while a PSA cut-off of 20.85 ng/mL would correctly identify 95% of all bone metastasis. For all the above cut-offs, combining with an ALP cut-off of 44.0 IU/L could lead to a correct identification of all metastatic cases (100.0%). It suggests that to avoid sending too many patients for routine bone scans, while also avoiding missing cases of metastasis, we can choose a PSA cut-off point of 20.85ng/mL and combine/support this with an ALP cut-off point of 44.0 IU/L in the subgroup with PSA values between 4.0 and 20.85 ng/mL (for all newly diagnosed, treatment-naïve, prostate cancer patients assessing care at our urology clinics). That way, we may correctly identify up to 100% of all patients at high risk of bone metastasis in our prostate cancer patients in Ghana.

Receiver operator characteristic curve analysis

This section presents the findings from two bivariate logistic regression models assessing the individual predictive ability of PSA category (PSAcat) and ALP category (ALPcat) on the likelihood of bone metastasis (METcat) among newly diagnosed prostate cancer patients.

PSAcat as a Predictor of Bone Metastasis

**Figure 4:**
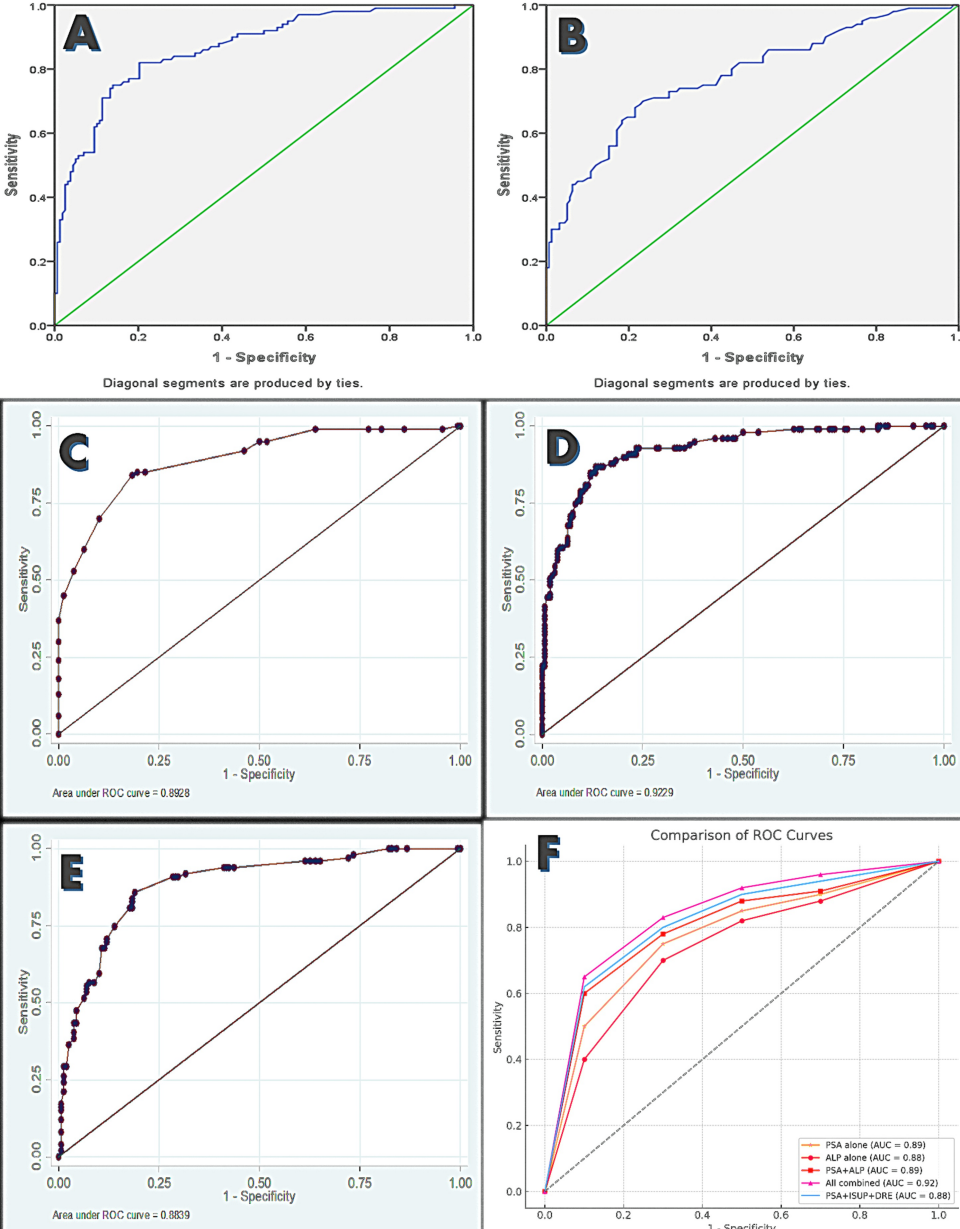
Comparative ROC analysis for models predicting bone metastasis in prostate cancer patients at Korle Bu Teaching Hospital This six-panel figure presents the diagnostic performance of various logistic regression models developed to predict the likelihood of bone metastasis in newly diagnosed prostate cancer patients, using serum biomarkers and clinical parameters. A: ROC curve for a model using serum PSA alone as the predictor. The AUC was 0.82, indicating good discriminatory power. B: ROC curve for a model using serum ALP alone, with an AUC of 0.88, suggesting comparable performance to PSA. C: ROC curve for a model combining PSA and ALP as predictors. This yielded a marginally improved AUC of 0.89, showing slight benefit over individual markers. D: ROC curve for a comprehensive model combining PSA, ALP, ISUP grade, and DRE risk. This model achieved the highest AUC of 0.92, reflecting excellent diagnostic accuracy and robustness. E: ROC curve for a simplified multiparametric model using PSA, ISUP grade, and DRE risk (excluding ALP). This model produced an AUC of 0.88, indicating good performance but slightly inferior to the full model. F: Combined ROC plot comparing all five models on a single axis. Each model’s ROC curve is color-coded with distinct markers and includes a legend for reference. The comprehensive model (PSA + ALP + ISUP + DRE) shows the highest curve, emphasizing the added predictive value of a multivariable approach. This combined visualization clarifies the benefit of incorporating multiple clinical and biochemical parameters, particularly ALP, ISUP grade, and DRE findings, into bone metastasis risk estimation among prostate cancer patients in resource-limited settings. ALP, alkaline phosphatase; PSA, prostate-specific antigen; ISUP, International Society of Urological Pathology grade group; DRE_Risk, disease risk level on digital rectal examination findings; AUC, area under the ROC curve; ROC, receiver operating characteristic curve

The logistic regression model using PSAcat alone demonstrated strong predictive value. PSAcat was significantly associated with bone metastasis, with an odds ratio (OR) of 4.59 (95% CI: 3.05-6.93, p < 0.001), indicating that patients in higher PSA categories had nearly five times the odds of having bone metastases compared to those in lower PSA categories. The model showed a pseudo R² of 0.2789 and a respectable AUC of 0.8165, reflecting good discriminatory power (Tables 5, 6, Figure 4).

**Table 5:** Bivariate predictors of bone metastasis in prostate cancer In the bivariate analysis, both PSAcat and ALPcat were significant individual predictors of bone metastasis, each demonstrating acceptable to strong performance. PSAcat showed superior overall discrimination (higher AUC and specificity), while ALPcat showed better sensitivity. Individually, ISUP and clinical stage at DRE provided AUCs (0.6973 and 0.7919, respectively) for predicting bone metastasis in treatment-naïve prostate cancer patients. These findings support the inclusion of the variables in multivariable predictive models for metastatic prostate cancer, especially in resource-limited settings where early risk stratification is critical. ALP, alkaline phosphatase; PSA, prostate-specific antigen; ISUP, International Society of Urological Pathology grade group; DRE_Risk, clinical risk level based on digital rectal examination findings; Cat, category; AUC, area under the ROC curve; ROC, receiver operating characteristic curve

Predictor	Odds Ratio	95% CI	p-Value	Sensitivity (%)	Specificity (%)	AUC (%)
PSAcat	4.59	3.05–6.93	<0.001	56	90.5	81.7
ALPcat	3.24	2.34–4.47	<0.001	70	76.6	77.9
ISUPcat	1.73	1.34–2.24	<0.001	61.7	84	76.7
DREcat	1.8	1.05–3.10	0.032	68.1	68.7	70.1

**Table 6:** Logistic regression models, model performance metrics, and predictor odds ratios ALP, alkaline phosphatase; PSA, prostate-specific antigen; ISUP, International Society of Urological Pathology grade group; DRE_Risk, clinical risk level based on digital rectal examination findings; %HistVol/volHist, the percentage volume of the tumor biopsy cores with adenocarcinoma involvement; PeriInv, perineural invasion; Cat, category; AUC, area under the ROC curve; ROC, receiver operating characteristic curve; TN, true negatives; FP, false positives; FN, false negatives; TP, true positives

Model	Pseudo R²	AUC	Sensitivity	Specificity	Accuracy	Precision	F1 Score	LLR/HL p-Value	PSA, OR (95% CI)	ALP, OR (95% CI)	DRE_Risk, OR (95% CI)	ISUP, OR (95% CI)	Confusion Matrix (TN, FP, FN, TP)
PSAcat + ALPcat	0.413	0.8928	0.7	0.899	0.822	0.814	0.7527	0.3006	4.26 (2.74–6.62)	3.16 (2.13–4.68)	N/A	N/A	142, 16, 30, 70
PSAcat + ALPcat + DREcat + ISUP	0.491	0.9229	0.788	0.899	0.856	0.8316	0.8103	0.9626	2.84 (1.78–4.52)	3.29 (2.15–5.04)	1.72 (0.98–3.02)	1.92 (1.42–2.61)	142, 16, 21, 79
PSAcat + DREcat + ISUP	0.364	0.8839	0.677	0.892	0.809	0.7976	0.7283	0.095	3.43 (2.22–5.30)	N/A	1.80 (1.05–3.10)	1.73 (1.34–2.24)	141, 17, 33, 67
DRE_Risk	0.1023	0.6973	0.6809	0.6867	0.6844	0.5766	0.6244	N/A	N/A	N/A	2.3959 (1.7494–3.2812)	N/A	103, 47, 30, 64
ISUP	0.2097	0.7919	0.617	0.84	0.7541	0.7073	0.6591	N/A	N/A	N/A	N/A	N/A	126, 24, 36, 58
ALP + DRE_Risk + ISUP	0.4049	0.8883	0.7234	0.8533	0.8033	0.7556	0.7391	N/A	N/A	1.0276 (1.0151–1.0402)	2.3851 (1.8836–3.0201)	N/A	128, 22, 26, 68
PSA non-categorized + ALPnon-categorized + ISUP + DRE_Risk	0.4376	0.9075	0.734	0.9067	0.8402	0.8313	0.7797	0	1.0024 (1.0001–1.0047)	1.0263 (1.0138–1.039)	1.5907 (1.0454–2.4206)	2.0018 (1.4889–2.6915)	136, 14, 25, 69
PSA + ALP + ISUP + DRE_Risk + bone pain	0.459	0.933	0.913	0.913	0.843	0.845	0.877	<0.0001	1.0022 (0.9999–1.0045)	1.03 (1.02–1.04)	1.58 (1.03–2.42)	2.2687 (1.7005–3.0269)	136/13/25/68
PSA + ALP + ISUP + DRE_Risk + bone pain + VolHist + PeriInv + cribriform	0.475	0.939	0.8245	0.913	0.846	0.844	0.832	<0.0001	1.002 (0.999–1.005)	1.029 (1.015–1.043)	1.666 (1.07–2.59)	N/A	TN:136, FP:13, FN:24, TP:67
ALP + ISUP + DRE_Risk + bone pain	0.429	0.909	0.8015	0.872	0.818	0.8105	0.8055	<0.0001	N/A	1.030 (1.017–1.044)	1.756 (1.16–2.67)	N/A	TN:130, FP:19, FN:25, TP:68
PSA + ISUP + DRE_Risk + bone pain	0.459	0.933	0.822	0.913	0.843	0.842	0.8295	<0.0001	1.002 (0.999–1.005)	1.029 (1.015–1.043)	1.580 (1.04–2.50)	2.0018 (1.4889–2.6915)	TN:136, FP:13, FN:25, TP:68

In terms of classification accuracy, the model correctly classified 77.13% of the cases. Sensitivity was 56.0%, suggesting moderate ability to correctly identify patients with metastases, while specificity was high at 90.51%, reflecting excellent performance in ruling out non-metastatic cases. The model passed the Hosmer-Lemeshow goodness-of-fit test (p = 0.9699), confirming good calibration.

ALPcat as a Predictor of Bone Metastasis

Similarly, the bivariate model with ALPcat alone as the predictor also showed statistical significance. ALPcat had an OR of 3.24 (95% CI: 2.34-4.47, p < 0.001), confirming that elevated ALP levels were associated with over three times the odds of having bone metastasis (Tables 5, 6, Figure 4). This model had a pseudo R² of 0.2138 and an AUC of 0.7797, suggesting acceptable discriminatory capacity but slightly lower than PSAcat. It correctly classified 74.03% of the patients, with a sensitivity of 70.0% and specificity of 76.6%. The model also passed the Hosmer-Lemeshow test (p = 0.3765), indicating an acceptable fit. The AUC for ISUPcat in predicting bone metastasis was 76.7%; while that for DREcat was 70.1% (Tables 5, 6).

Multivariate analysis and model building

Three logistic regression models were fitted to predict bone metastasis (METcat) in prostate cancer patients based on combinations of PSAcat, ALPcat, DREcat, and ISUP score. The first model (PSAcat and ALPcat combined) showed that both PSAcat (aOR: 4.26, p < 0.001) and ALPcat (aOR: 3.16, p < 0.001) were significant predictors, with a pseudo R² of 0.413 and AUC of 0.8928. The model correctly classified 82.2% of patients, with a sensitivity of 70% and specificity of 89.9%. The Hosmer-Lemeshow goodness-of-fit test was acceptable (p = 0.30).

The second model, which added DREcat and ISUP score, had improved discrimination with an AUC of 0.9229 and a higher Pseudo R² of 0.491. ALPcat (adjusted odds ratios [aOR]: 3.29), PSAcat (aOR: 2.84), and ISUP (aOR: 1.92) were statistically significant predictors (p < 0.001), while DREcat approached significance (p = 0.061). This model achieved an accuracy of 85.6%, sensitivity of 78.8%, and specificity of 89.9%. The Hosmer-Lemeshow test indicated excellent fit (p = 0.963).

The third model, excluding ALPcat but retaining PSAcat, DREcat, and ISUP, showed decreased performance, with an AUC of 0.8839 and pseudo R² of 0.364. PSAcat (aOR: 3.43), DREcat (aOR: 1.80), and ISUP (aOR: 1.73) remained significant (p < 0.05). Classification accuracy dropped to 80.9%, with lower sensitivity (67.7%) but similar specificity (89.2%).

Across models, PSAcat and ISUP consistently emerged as robust predictors of metastasis. The inclusion of ALPcat improved both discrimination and calibration, suggesting that bone metabolism markers enhance predictive accuracy.

The findings in Table 6 summarize the performance and odds ratios of four logistic regression models (plus 6 others incorporating additional predicting parameters namely, bone pain, cribriform pattern, perineural invasion, and percentage of core biopsy involved with tumor, in various combinations) predicting bone metastasis using various combinations of clinical and pathological variables. Key metrics such as AUC, pseudo R², accuracy, and statistical significance were considered (Tables 5-7, Figure 4).

**Table 7:** Comparison of predictive models for bone metastasis using AUC and Z-statistics (for the hypothesis testing) This table summarizes the stepwise performance improvement of eight logistic regression models developed to predict bone metastasis (METcat) in prostate cancer patients at Korle Bu Teaching Hospital. Each model incorporates additional clinical or biochemical variables to assess their contribution to overall diagnostic accuracy. The models are compared using the AUC from ROC analysis, SE, and Z-statistics to test the statistical significance of AUC differences between consecutive models. A critical Z-value of ±1.96 (α = 0.05) was used as the threshold for statistical significance. Statistically significant improvements were observed upon the addition of ALPcat (model 2) and further upon inclusion of ISUP and DRE_Risk (model 4), while model 3 did not offer a significant improvement over model 2. Further evaluation revealed that model 4 showed modestly better classification (net reclassification improvement = 0.272) and demonstrated consistently higher net clinical benefit than model 3 across threshold probabilities of 0.15 to 0.65 on decision curve analysis. ALP, alkaline phosphatase; PSA, prostate-specific antigen; ISUP, International Society of Urological Pathology grade group; DRE_Risk, clinical risk level based on digital rectal examination findings; %HistVol/volHist, percentage volume of the tumor biopsy cores with adenocarcinoma involvement; PeriInv, perineural invasion, Cat, category; Met, metastasis; AUC, area under the ROC curve; ROC, receiver operating characteristic curve

Model Comparison	Model Independent Parameters	AUC	Standard Error	Change in Z-Statistic	Critical Z-Value (α = 0.05)
Model 1: METcat vs. PSAcat	PSAcat	0.8165	0.0254	-	±1.96
Model 2: METcat vs. PSAcat + ALPcat	PSAcat + ALPcat	0.8928	0.019	-2.41 (vs. Model 1)	±1.96
Model 3: METcat vs. PSAcat + DREcat + ISUP	PSAcat + DREcat + ISUP	0.8743	0.0186	-0.65 (vs. Model 2)	±1.96
Model 4: METcat vs. PSAcat + ALPcat + DREcat + ISUP	PSAcat + ALPcat + DREcat + ISUP	0.9229	0.0186	-1.85 (vs. Model 3)	±1.96
Model 4 vs. Model 2	PSAcat + ALPcat + DREcat + ISUP	0.9229	0.0186	+1.13 (vs. Model 2)	±1.96
Model 7 vs. Model 4	PSA + ALP + ISUP + DRE_Risk + Bone pain	0.933	0.0186	0.38	±1.96
Model 8 vs. Model 4	PSA + ALP + ISUP + DRE + Bone pain + %HistVol + PeriInv + Cribriform	0.939	0.0186	0.61	±1.96
Model 9 vs. Model 4	ALP + ISUP + DRE + Bone pain	0.909	0.0186	-0.53	±1.96
Model 10: vs. Model 4	PSA + ISUP + DRE + Bone pain	0.933	0.0186	0.38	±1.96

The net reclassification improvement (NRI) analysis comparing model 4 (PSAcat + ALPcat + DREcat + ISUPcat) and model 3 (PSAcat + DREcat + ISUPcat) showed that model 4 modestly improved overall risk classification, with an NRI of 0.272. This improvement was primarily driven by better classification of non-events despite a slight decline in reclassification accuracy for true events. Furthermore, decision curve analysis (DCA) demonstrated that model 4 consistently provided greater net clinical benefit than model 3 across threshold probabilities between 0.15 and 0.65. These findings support the added value of including ALPcat in the model to enhance clinical decision-making.

Summary of hypothesis testing and model comparison

Based on the hypothesis set at the outset of this study, we comparatively evaluated the predictive power of several logistic regression models for bone metastasis in prostate cancer using AUC and Z-statistics to assess model improvements (Tables 7, 8).

**Table 8:** Hypothesis testing decisions for the bone metastasis predictive models ALP, alkaline phosphatase; PSA, prostate-specific antigen; ISUP, International Society of Urological Pathology grade group; DRE_Risk, clinical risk level based on digital rectal examination findings; PeriInv, perineural invasion; %HistVol, percentage volume of the tumor biopsy cores with adenocarcinoma involvement; Cat, category; AUC, area under the ROC curve; ROC, receiver operating characteristic curve

Hypothesis	Statement	Statistical Result	Conclusion on Hypothesis
H1	Increasing total serum PSA is associated with a higher likelihood of bone metastasis.	Model 1 shows good discrimination (AUC = 0.8165). OR of 4.59 (95% CI: 3.05–6.93, p < 0.001) for PSA.	Accepted (failed to reject)
H2	Elevated serum ALP is associated with an increased risk of bone metastasis.	OR of 3.24 (95% CI: 2.34–4.47, p < 0.001) for ALP.	Accepted (failed to reject)
H3	The combined predictive value of ALP and PSA is superior to PSA alone.	Model 2 vs. model 1 shows significant AUC improvement from 0.8165 to 0.8928, Z = -2.41.	Accepted (failed to reject)
H4	Higher DRE clinical stage and ISUP grade correlate with increased bone metastasis risk.	Mets vs DRE and Mets vs ISUP. Models show AUCs = 0.6973 and 0.8991, respectively. DREcat (OR: 1.80) and ISUP (OR: 1.73) both significant (p < 0.05).	Accepted (failed to reject)
				H5	The combined predictive value of DRE, ISUP, ALP, and PSA is greater than PSA and ALP together.	Model 4 vs. model 2 has Z = +1.13 (not significant). There was a considerable increase but not up to 1.96, which is the critical value.	Rejected (statistically rejected but may show clinically useful marginal benefits)
H6	Comprehensive model (PSA, ALP, DRE, ISUP, bone pain, %HistVol, PeriInv, cribriform) is superior to the base model.	Model 8 vs. model 4, Z = +0.61 (not significant).	Rejected (statistically rejected but may show clinically useful marginal benefits)

Based on Tables 7, 8, we conclude that based on statistical analysis, as well as a follow-up NRI and DCA, model 4 and model 8 offer the best performances. Based on practical considerations, Model 4 offers the best trade-off between performance and practicality (AUC = 0.9229), using routinely available clinical parameters. It is the recommended model for predicting bone metastasis, and thus we proceed with it. The final selected model for this study is therefore the model involving Mets vs PSAcat, ALPcat, DREcat, and ISUP.

Model internal validation, training, and external validation: summary and comparison

The study data were partitioned into three sets of 70% (as model development dataset), 10% (as model training data), and 20% (as model external validation dataset).

With the development dataset, a multivariable logistic regression model was developed to predict the likelihood of bone metastasis among prostate cancer patients using four predictors: PSA, serum ALP, ISUP grade, and DRE-based clinical risk grouping. The model equation derived from the development dataset was as follows:

\[ \text{Logit}(P) = -2.4693 + 0.0065 \cdot \text{PSA} + 0.0063 \cdot \text{ALP} + 0.6456 \cdot \text{ISUP} + 0.7462 \cdot \text{DRE}_{\text{Risk}} \]

The model demonstrated excellent performance during development, with an AUC of 0.902, accuracy of 88.1%, sensitivity of 84.0%, and specificity of 90.9%. The pseudo R² of 0.518 and highly significant LLR p-value (<0.0001) indicated good model fit. Multicollinearity was assessed using variance inflation factors (VIFs) for all predictors (PSA, ALP, ISUP, and DRE Risk), each of which fell well below the threshold of 10 (range: 1.05-1.21), confirming the absence of problematic multicollinearity. This supports the model’s stability and reliability for inference and prediction.

Upon training with an independent 10% subset of the data, the model retained good predictive ability (AUC: 0.86), with accuracy of 87.5%, sensitivity of 77.8%, and specificity of 93.3%. The training model equation adjusted slightly to:

\[ \text{Logit}(P) = -5.3134 + 0.0074 \cdot \text{PSA} + 0.0108 \cdot \text{ALP} + 0.7527 \cdot \text{ISUP} + 0.4239 \cdot \text{DRE}_{\text{Risk}} \]

**Figure 5:**
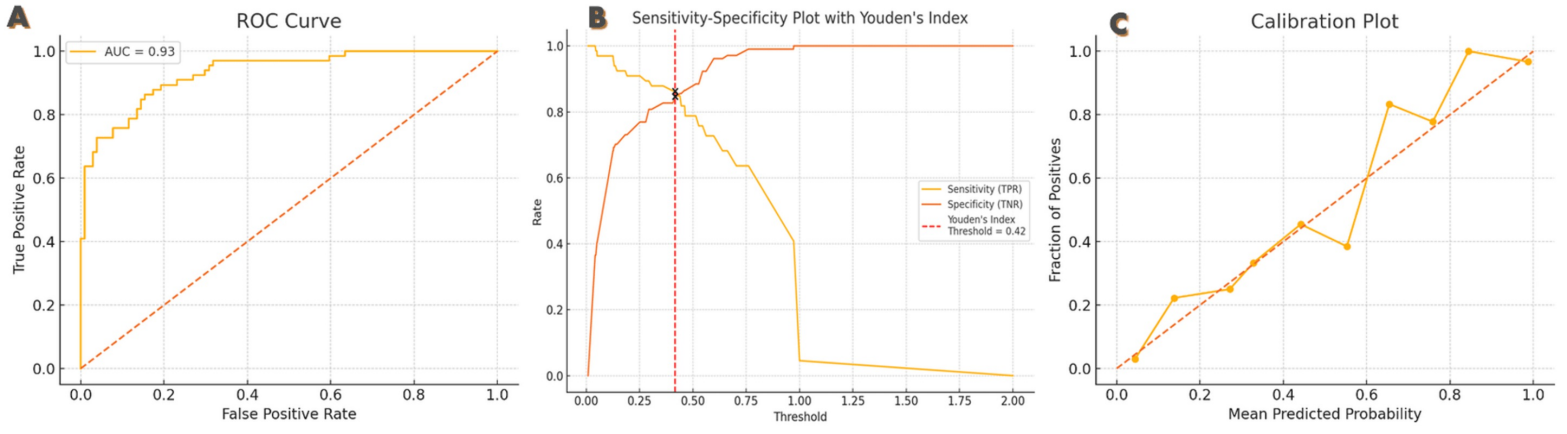
Internal validation performance of the multiparametric logistic regression model for predicting bone metastasis in treatment-naïve prostate cancer patients using PSA, ALP, ISUP grade, and DRE risk in a Ghanaian cohort This composite figure illustrates the internal validation metrics of a multivariable logistic regression model developed to predict the likelihood of bone metastasis in newly diagnosed prostate cancer patients. The model incorporated total serum PSA, serum ALP, ISUP grade, and DRE risk classification as predictors. Data were derived from prostate cancer patients. A (ROC curve): the ROC curve demonstrates strong discriminatory performance of the model, with an AUC of 0.93. The curve plots the true-positive rate (sensitivity) against the false-positive rate (1-specificity) across all probability thresholds, indicating excellent model accuracy in distinguishing patients with and without bone metastasis. B (sensitivity-specificity plot): this plot presents the sensitivity and specificity trends across various decision thresholds, with Youden’s Index (J = sensitivity + specificity – 1) used to determine the optimal threshold for classification. The vertical red dashed line marks the optimal cutoff probability (threshold = 0.42) at which the balance between true positives and true negatives is maximized. This threshold may serve as a decision point for recommending further staging investigations (e.g., bone scans). C (calibration plot): the calibration plot compares the predicted probabilities from the model to the actual observed outcomes (fraction of patients with bone metastasis). The closer the calibration curve is to the 45-degree diagonal reference line (ideal calibration), the better the agreement between predicted and observed risks. The model demonstrates good calibration across most risk bins, confirming reliability in absolute risk estimation. This figure provides internal validation evidence for the predictive model and supports its potential utility in resource-limited settings. PSA, prostate-specific antigen; ALP, alkaline phosphatase; ISUP, International Society of Urological Pathology grade group; DRE, digital rectal examination

**Figure 6:**
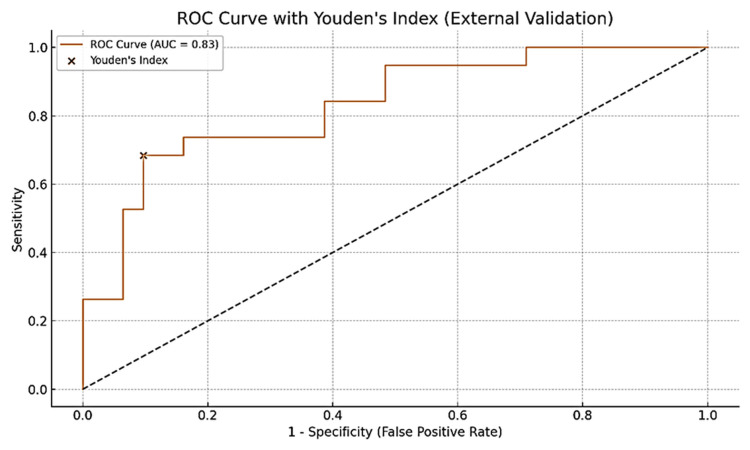
ROC curve with the new adjusted Youden’s index following external validation of the bone metastasis prediction model This figure presents the ROC curve generated from the external validation dataset for the logistic regression model predicting bone metastasis in prostate cancer patients using a combination of clinical and biochemical parameters. The ROC curve plots the model’s sensitivity (true positive rate) against 1-specificity (false-positive rate) across a range of probability thresholds. The model post-external validation demonstrated an AUC of 0.83, indicating good discriminatory power in an independent patient cohort not used during model development. The Youden’s Index (J = sensitivity + specificity – 1, which comes to 0.68 + 0.92 – 1 = 0.60) is marked with a cross (×) on the curve, identifying the optimal threshold that maximizes both sensitivity and specificity (it calculates to 0.60). This threshold is clinically relevant for decision-making on whether to proceed with bone scan imaging. The dashed diagonal line represents the line of no discrimination (AUC = 0.5), while the model’s ROC curve lies well above this line, confirming its usefulness in stratifying metastatic risk. This external validation supports the model’s generalizability and potential for application in broader clinical settings, particularly within resource-limited environments such as sub-Saharan Africa. AUC, area under the ROC curve; ROC, receiver operating characteristic curve

External validation using a held-out 20% unseen dataset confirmed acceptable generalizability, with an AUC of 0.793, accuracy of 70.0%, sensitivity of 68.4%, specificity of 70.9%, PPV of 59.1%, and NPV of 78.6%. In this round, the development model equation was applied directly during external validation without modification (Table 9, Figures 5, 6).

**Table 9:** Model internal validation, training, and external validation: summary and comparison of model metrics with model equations at each stage ALP, alkaline phosphatase; PSA, prostate-specific antigen; ISUP, International Society of Urological Pathology grade group; DRE_Risk, disease risk level based on Digital Rectal Examination findings; Cat, category; Mets, metastasis; AUC, area under the ROC curve; ROC, receiver operating characteristic curve; NPV, negative predictive value

Stage	Resulting Model Equation at Each Stage	Accuracy	Sensitivity	Specificity	Precision	NPV	F1 Score	False-Positive Rate	False-Negative Rate	AUC
Development	Logit(P) = -2.4693 + 0.0065×PSA + 0.0063×ALP + 0.6456×ISUP + 0.7462×DRE_Risk	0.8814	0.84	0.9085	0.8077	0.9333	0.8231	0.0915	0.16	0.9024
Training	Logit(P) = -5.3134 + 0.0074×PSA + 0.0108×ALP + 0.7527×ISUP + 0.4239×DRE_Risk	0.875	0.7778	0.9333	0.875	0.875	0.8235	0.0667	0.2222	0.86
External validation	Same model equation from training stage used for prediction	0.7	0.6842	0.7097	0.5909	0.7857	0.6341	0.2903	0.3158	0.7929
Model (second round)	Equation (second round)	AUC	Optimal threshold	Accuracy	Precision	Recall (sensitivity)	Specificity	F1 score	Brier score	Log loss
Baseline model (external validation)	Logit(P) = –5.3134 + 0.0074·PSA + 0.0108·ALP + 0.7527·ISUP + 0.4239·DRE_Risk	0.8217	0.6622	0.82	0.8125	0.6842	0.9062	0.7429	0.1292	0.3545
Final combined model (reunified dataset)	Logit(P) = -6.3587 + 0.0024·PSA + 0.0260·ALP + 0.6941·ISUP + 0.4642·DRE_Risk	0.9075	0.2999	0.8197	0.7232	0.8617	0.7736	0.7863	0.1184	0.3176

These results support the model’s robustness in estimating the probability of bone metastasis using routinely available clinical parameters and demonstrate its potential for integration into clinical decision-making frameworks or digital prediction tools (Table 9, Figures 5, 6).

The model equation post-validation is as follows:

\[ \text{Logit}(P) = -5.3134 + 0.0074 \cdot \text{PSA} + 0.0108 \cdot \text{ALP} + 0.7527 \cdot \text{ISUP} + 0.4239 \cdot \text{DRE}_{\text{Risk}} \]

**Figure 7:**
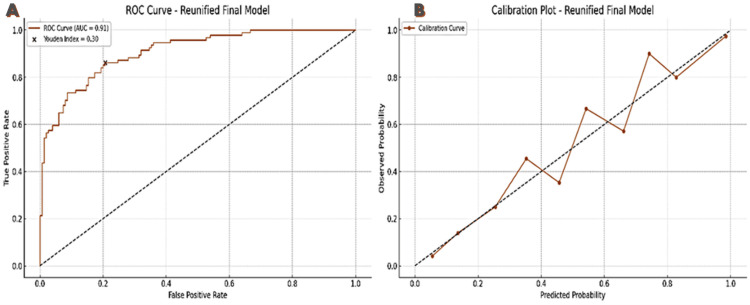
Discrimination and calibration performance of the final logistic regression model The image shows the combined performance plots of the final updated logistic regression model predicting bone metastasis in prostate cancer patients. A. ROC curve showing excellent discrimination (AUC = 0.91) with the optimal Youden’s Index threshold (0.30) marked. B. Calibration plot demonstrating close agreement between predicted and observed probabilities across deciles. AUC, area under the ROC curve; ROC, receiver operating characteristic curve

The training and retraining of the model continued iteratively. The final training and retraining of the internally and externally validated logistic regression model (Figure 7) were done using the full dataset available (development and external validation dataset together [29, 30]) and demonstrated excellent predictive performance for identifying bone metastases in treatment-naïve prostate cancer patients. The equation Logit(P) = -6.3587 + 0.0024·PSA + 0.0260·ALP + 0.6941·ISUP + 0.4642·DRE_Risk achieved an AUC of 0.91, with a sensitivity of 86.2%, specificity of 77.4%, and an overall accuracy of 81.9% at the optimal Youden threshold (0.30). The model also showed strong calibration (Brier score = 0.118) and a low log loss (0.318), affirming its robustness and clinical utility for metastatic risk stratification.

The final equation post-training is as follows:

\[ \text{Logit}(P) = -6.3587 + 0.0024 \cdot \text{PSA} + 0.0260 \cdot \text{ALP} + 0.6941 \cdot \text{ISUP} + 0.4642 \cdot \text{DRE}_{\text{Risk}} \]

Digital risk calculator

The final validated and trained model was then programmed into a digital risk calculator using R Platform for statistical analysis. The interface is shown in Appendix A. Usability testing is being conducted using a structured 5-point Likert-scale tool with clinical users in a preliminary study.

One may follow the following link to access the online version of the interactive digital risk estimator for bone metastasis in prostate cancer patients: https://raw.githack.com/qwabsfiles/healthtools/main/kbth.html

The questionnaire for this study is presented as a table in Appendix B.

## Discussion

Key findings

This study confirms that total serum PSA and ALP are strong predictors of bone metastasis in newly diagnosed, treatment-naïve prostate cancer patients in Ghana. Among 258 men, PSA alone showed excellent performance (AUC = 81.65%), and combining it with ALP improved sensitivity and risk stratification. Practical cut-off values of 18.95 ng/mL for PSA and 59.48 IU/L for ALP can help exclude low-risk individuals from unnecessary staging bone scans. Other significant predictors included DRE stage, ISUP grade, perineural invasion, cribriform histology, percentage of the core of prostate biopsy involved with adenocarcinoma, and bone pain, all of which enhanced multivariable models, though PSA remained the most robust standalone marker. ALP, while useful at baseline, showed no significant short-term changes post-treatment, limiting its value for early response monitoring. In this cohort, a PSA cut-off of 10.0 ng/mL identified 99% of metastasis, while 18.95 ng/mL and 20.85 ng/mL identified 97.5% and 95%, respectively. Crucially, combining any of these with an ALP cut-off of 44.0 IU/L correctly identified 100% of metastatic cases. Therefore, adopting a PSA threshold of 20.85 ng/mL - supplemented by ALP testing for those within the 4.0-20.85 ng/mL range - could allow accurate identification of all high-risk patients while reducing unnecessary bone scans.

Comparison with literature

This study highlights the independent and complementary predictive roles of serum PSAcat and ALPcat in assessing the likelihood of bone metastasis (METcat) among newly diagnosed, treatment-naïve prostate cancer patients in Ghana. In bivariate logistic regression, PSAcat showed a strong association with metastasis (OR = 4.59), high specificity (90.5%), and a robust AUC of 0.8165, reaffirming its central role in prostate cancer staging [8,15,20,28]. ALPcat, though less specific, demonstrated higher sensitivity (70.0%) and significant predictive power (OR = 3.24, AUC = 0.7797), supporting its known link to osteoblastic activity and skeletal involvement [23,24]. Both models were well-calibrated (Hosmer-Lemeshow p-values: PSAcat = 0.9699; ALPcat = 0.3765), though PSAcat exhibited higher classification accuracy and pseudo R².

Both biomarkers demonstrated statistically significant associations with metastases and contributed to distinct strengths, with PSA providing high specificity and overall discrimination and ALP enhancing sensitivity and early detection and helping reduce false negatives.

With these, PSA remains a cornerstone in prostate cancer staging and these findings align with prior studies linking PSA levels >10-20 ng/mL to increased metastatic potential [8,12,17,25].

In parallel, ALPcat performance is consistent with its role as a marker of osteoblastic activity and skeletal involvement in malignancies [23,24]. While less specific than PSA, ALP showed higher sensitivity (70.0% vs. 56.0%), highlighting its clinical utility in minimizing false negatives and improving triage sensitivity [23,24,26].

These findings are especially pertinent in sub-Saharan Africa, where advanced-stage presentation is common and access to imaging such as bone scintigraphy is often limited [1,4,6,9,10,14]. Several West African studies have reported frequent metastatic disease even at intermediate PSA levels [1,9,10,14,17,24] due to delayed presentation, limited screening programs, and biologically aggressive tumor variants in African men [9,10,14,16-22].

Incorporating PSAcat and ALPcat into multivariable models significantly improved overall discrimination. The best-performing model (PSAcat + ALPcat + DREcat + ISUP) achieved an AUC of 0.9229, classification accuracy of 85.6%, and strong calibration (Hosmer-Lemeshow p = 0.9626), confirming its diagnostic robustness. PSAcat remained the most consistent predictor, while ISUP grade (particularly ≥3) also significantly contributed to metastatic prediction [3,12,19,20,25].

The impact of ALPcat was evident in the increase in AUC from 0.8839 to 0.9229 when added to the multivariable model (involving PSA, ISUP, and DRE). This is in line with previous findings [23,24]. Disease stage by DRE, although not independently significant (p = 0.061), improved model classification and remain clinically useful, [3,12,25]. However, the observation that another similar model (Mets vs PSAcat, DREcat, ISUPcat, and bone pain) also yielded an AUC of 0.933 in this study suggests that for this population, the ideal model for predicting bone metastasis may not be singular.

The metastasis rate of 38.8% observed in this cohort reflects a broader pattern across African countries, where high-risk diseases are frequently diagnosed at presentation [1,4-7,14]. Contributing factors may include genetic predisposition and molecular features such as elevated TMPRSS2:ERG fusions and aggressive tumor biology [1,20]. This makes the extended model derived in this study (that incorporates the eight parameters: PSAcat, ALPcat, DREcat, ISUP, perineural invasion, cribriform histology, percentage of the core prostate biopsy involved with adenocarcinoma, and bone pain) a potentially a good model for further study and validation with larger datasets going forward. It also holds promise since it displayed an AUC of up to 93.90% and specificity and sensitivities of 91.30% at the outset, though these need to be re-evaluated and are beyond the scope of this paper.

Importantly, this study proposes locally derived, pragmatic PSA and ALP thresholds. A PSA cut-off of 10.0 ng/mL identified 99.0% of metastases, while 18.95 ng/mL and 20.85 ng/mL identified 97.5% and 95.0%, respectively. Combining any of these PSA thresholds with ALP ≥44.0 IU/L achieved 100.0% detection. Thus, using PSA ≥20.85 ng/mL plus ALP ≥44.0 mmol/L (in the subgroup with a PSA 4.0-20.85 ng/mL) could provide an effective, safe, and cost-conscious strategy for triaging patients for bone scans in resource-limited environments. These values align with international guidelines [3,12,17,25] yet are tailored for West African populations, where metastasis may occur at lower PSA levels [9,12,14,20].

ALP’s limited short-term variability over three months post-diagnosis suggests that it is not suitable for early treatment response monitoring [23,24]. Rather, its value lies in baseline staging, reflecting cumulative osteoblastic burden rather than real-time tumor activity in prostate cancer, unlike PSA.

The findings of this study add to a growing body of literature validating PSA as a central marker in metastasis prediction. Zaman et al. showed safe exclusion of metastases in Pakistani men with PSA ≤20 ng/mL and Gleason <8 [11], Wei et al. identified a 13 ng/mL threshold in Taiwanese patients [22], and Kamaleshwaran et al. found >97% of Indian men with PSA <20 ng/mL had no bone metastases [16]. This study extends such evidence by offering prospectively derived PSA and ALP thresholds specific to West African populations.

Though often used as a confirmatory marker, ALP's utility in combination with PSA is reaffirmed here, particularly where access to imaging is constrained [26,27]. The multivariable model reflects global staging frameworks such as the D’Amico classification [3,12,25], yet it is calibrated to local epidemiology, avoiding over- or under-utilization of diagnostic resources. Such may support previous calls for locally informed models tailored to West African populations, where molecular, epidemiological, and access-related differences limit direct extrapolation of Western guidelines [1,5,14,20]. Additionally, this study contributes to the broader effort of improving guideline adaptation for prostate cancer staging in low- and middle-income countries, in line with evolving recommendations from both AUA and EAU [3,12,25].

Concisely, PSAcat and ALPcat are effective and synergistic tools for predicting bone metastases in Ghanaian prostate cancer patients. Their integration into locally validated multiparametric models offers a practical, cost-effective approach to improving staging decisions. Future studies should focus on external validation across West Africa and explore molecular markers (e.g., TMPRSS2:ERG, PCA3) to further enhance accuracy [20].

Model finalization: internal validation, external validation, re-calibration/updating

Having selected the optimal model after the hypothesis testing and practical considerations, we proceeded to develop it further, train it, and internally and externally validate it for potential application in risk estimation of bone metastasis in newly diagnosed prostate cancer patients using PSA, serum ALP, ISUP grade, and DRE-based clinical risk classification.

The modelling process involved sequential development, refinement, and validation of a logistic regression-based risk estimator for predicting bone metastasis in newly diagnosed prostate cancer patients using four routinely available variables: PSA, ALP, ISUP grade, and DRE-based clinical risk. The model underwent development, internal development, training, external validation, and retraining/updating using the unified data [29,30]. This modelling approach yielded a well-calibrated and externally validated risk estimation tool with high clinical applicability, particularly in low-resource settings where bone scans may not be readily accessible. Its design, validation strategy, and performance metrics reflect adherence to robust modelling principles and real-world applicability [29,30].

The initially developed model demonstrated high performance with an AUC of 0.9024, accuracy of 88.1%, sensitivity of 84%, and specificity of 90.9%. These metrics suggest strong discriminatory capacity and clinical utility, particularly given the high NPV (93.3%) - a desirable feature for ruling out metastatic disease.

On internal validation and training, the slightly adjusted model equation yielded an AUC of 0.86, with balanced sensitivity (77.8%) and specificity (93.3%). The precision and NPV remained high (87.5% each), indicating retained model robustness. The minor drop in sensitivity likely reflects an overfit correction during regularization and optimization [29,30].

External validation using the same initial model equation saw performance metrics decline as expected - a common occurrence when models are applied to unseen data according to Siontis et al. [29,30]. The AUC dropped to 0.7929, sensitivity to 68.4%, and specificity to 70.9%, though the model still achieved a reasonable NPV of 78.6%. This reaffirms the importance of external validation to assess generalizability. However, external validation of the trained initial model equation, as baseline, yielded an AUC of 0.8217 and balanced performance (accuracy 82%, F1 score 0.7429). Further adjustment led to an updated model with modified coefficients, suggesting recalibration to suit a new population context.

Final Model Training and Updating Using Unified Dataset

The finalized model, retrained on the full combined dataset (development plus validation data together), produced a high AUC (0.9075), precision (72.3%), and NPV (86.2%), and improved calibration (lowest log loss: 0.3176). The optimal probability threshold was determined to be 0.30, suggesting that the model’s sensitivity was maximized at a lower cut-off - practical in screening scenarios where false negatives carry significant risk [29,30]. It also reflected consistency across datasets.

Statistical Rigor and Modelling Justification

Throughout, metrics such as the Brier score (0.1184) and log loss (0.3176) confirmed model calibration and reliability. No multicollinearity was detected (VIFs all <1.3), ensuring coefficient stability. The model’s progression aligns with best practices in predictive modelling, including multi-stage validation, recalibration, and external testing, as advocated by Steyerberg in their seminal work on clinical prediction models [29,30].

This final model is what has been programmed using R (R Foundation for Statistical Computing, Vienna, Austria) into the digital risk estimator for bone metastasis in Ghana, as introduced by this study. While our model’s strength lies in its simplicity and generalizability, future research may explore dynamic or machine-learning approaches incorporating additional biomarkers, genomic profiles, or longitudinal treatment data [10,16,20]. Nonetheless, this foundational model represents a pragmatic step toward risk-adapted staging and metastatic screening in high-burden regions, like Ghana.

What is known about this topic

Prostate cancer represents the leading genitourinary malignancy in Ghana and is among the top causes of male cancer mortality [4,6,7]. Diagnosis and management rely heavily on PSA, which has become a ubiquitous biomarker in urologic oncology [3,5]. Bone is the most common site of prostate cancer metastasis, often detected via 99mTc-MDP bone scans [17,18]. However, there is no consensus regarding universal bone scan use in all newly diagnosed patients. In resource-constrained environments, routine scanning is often impractical due to limited availability [26], high cost, and the physical burden placed on frail patients.

Furthermore, racial and ethnic differences have been noted in prostate cancer biology, presentation, and biomarker kinetics. African and African-descent men tend to present with more aggressive disease and at younger ages [1,5,9,14], yet population-specific data to guide clinical decisions have remained scarce.

What this study adds

This study is the first in Ghana to prospectively evaluate and validate PSA and ALP as predictors of bone metastasis among treatment-naïve patients. It provides data-driven, percentile-derived cut-off values for PSA and ALP, making it possible to exclude a significant proportion of low-risk patients from bone scans. For instance, using a PSA threshold of 18.95 ng/mL, up to 20% of newly diagnosed patients could avoid bone scanning while missing only 2.5% of metastatic cases.

Moreover, the study integrates clinical and histopathologic variables to build a composite risk model that outperforms individual predictors. The deployment of this model in a mobile app further enhances its clinical utility by offering real-time, bedside risk assessment. This innovation potentially bridges the gap between evidence and practice, especially in settings where decision support tools are often lacking.

Additionally, the study’s examination of ALP dynamics post-treatment adds new understanding to the limitations of this biomarker in early response assessment, guiding clinicians toward better follow-up strategies.

Strengths and limitations

Among the major strengths of this study is its prospective design, which minimizes recall and selection biases inherent in retrospective analyses. The use of standardized protocols for diagnosis, laboratory assessment, and imaging enhances data reliability. Furthermore, the incorporation of histopathologic and radiologic findings allows for a multidimensional analysis of metastatic risk.

Another strength is the study’s direct applicability; the findings are embedded in a digital clinical tool tailored to the local health system and ready for real-world implementation. This is particularly relevant in sub-Saharan Africa, where healthcare infrastructure limitations require practical, locally validated solutions.

However, the study has several limitations. Being a single-center study, the generalizability of the findings to other parts of Ghana or Africa may be limited. The lack of centralized histopathology review introduces the possibility of inter-observer variability in grading. Additionally, the three-month follow-up may not capture longer-term trends in biomarker dynamics, particularly for ALP. Imaging limitations also precluded comparison with newer modalities such as PET/CT or whole-body MRI, which are considered superior in high-resource settings [15].

## Conclusions

PSA and ALP exhibited strong independent associations versus bone metastasis, with PSA outperforming other predictors (AUC = 81.650%). PSA and ALP combined outperformed PSA alone or a combined PSA, ISUP, and DRE model. Finally, PSA, ALP, ISUP, and DRE combined in a single model outperformed all the former in bone metastasis prediction in prostate cancer. Metastasis in patients with cribriform pattern at histology was uniformly present. For categorized cut-offs, a PSA cut-off of 18.95 ng/mL or an ALP cut-off of 59.48 IU/L, individually, detected 97.5% of bone metastasis. Combining PSA >20.95 ng/mL and ALP >44 IU/L proved superior (100% detection rate). A PSA above 20 ng/mL, DRE >T2c, and Gleason score >7 identified 95% of bone metastasis. For high-risk diseases per D'Amico stratification, bone scans are advisable. Adopting a PSA cut-off of 18.95 ng/mL can exclude 20% of patients from unnecessary bone scans while missing only 2.5% of bone metastasis, translating into 170 USD of financial savings per client and 55,354 USD of national savings per annum. ALP enhances prediction but may not serve as a follow-up marker. In Ghana, clinicians may safely order a bone scan with a PSA cut-off of 18.95 ng/mL.

The implementation of these findings into a mobile-based risk calculator positions this work at the intersection of epidemiologic research, digital health innovation, and real-world clinical utility. In a resource-limited setting where judicious use of diagnostics is critical, this study lays the groundwork for cost-effective, evidence-based, and personalized prostate cancer care. It could also help bridge the equity gap for most of the tertiary urology centers outside the capital city of Ghana, where the quickest and most accessible and available form of imaging to detect bone metastasis in prostate cancer patients is still conventional X-rays of the lumbosacral spine, which is fraught with the limitations of poor sensitivity and specificity.

## Figures and Tables

**Figure 8:**
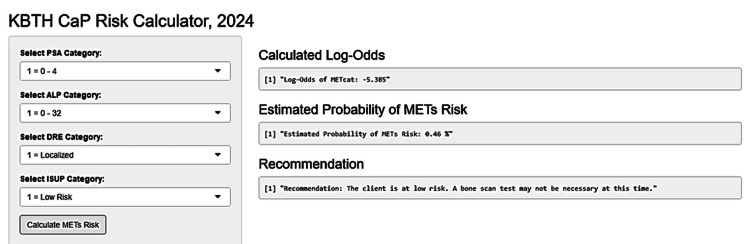
Interface of the KBTH CaP Digital Risk Calculator for estimating bone metastasis risk in prostate cancer patients This figure presents the user interface of the Korle Bu Teaching Hospital CaP Risk Calculator, a digital clinical decision support tool developed in 2024 to estimate the likelihood of skeletal metastases in patients newly diagnosed with prostate cancer. The calculator uses a multivariable logistic regression model integrating four key clinical parameters: PSA category (e.g., 0–4 ng/mL), ALP category (e.g., 0–32 IU/L), DRE category (e.g., localized vs. locally advanced), ISUP grade category (e.g., low risk, intermediate risk, high risk). Upon selecting values for each input category, the calculator computes and displays the following: calculated log-odds of bone metastasis, estimated probability of metastasis (expressed as a percentage), and a recommendation, which interprets the estimated probability in clinical context - advising whether or not a bone scan may be necessary. In the illustrated example, the selected combination of clinical parameters results in a very low estimated risk (0.46%) of bone metastasis. Accordingly, the tool generates a recommendation that a bone scan may not be necessary at this time. This digital platform is intended to assist clinicians in making evidence-informed decisions, especially in resource-constrained settings where bone scan access is limited. The interactive and active version of this tool is loaded as part of this manuscript submission as an interactive media. https://raw.githack.com/qwabsfiles/healthtools/main/kbth.html

## Appendices

Appendix A

Appendix B 

**Table 10:** Study questionnaire

Question	Subject 1
Serial no	
Referred from hospital	
Age	
Sex	
Occupation	
Educational level	
Ethnicity/tribe	
Nationality	
Family history of prostate cancer (father/brother/uncle)	
Smoking (yes/no); if yes, pack-years	
Alcohol intake (yes/no); if yes, quantity/week	
Bone pain/tenderness (yes/no)	
Current drugs or drug history	
PSA (ng/mL)	
Date of PSA test	
Prostate volume (mL) by TRUS-P	
DRE grade	
Clinical stage	
Histopathology: Gleason score	
Number of cores involved	
Percentage of cores involved	
% Volume of prostate involved	
ISUP grade / NCCN Risk Group (1–5)	
Bone scan findings (no mets / mets / equivocal)	
Sites of bone metastasis	
Bone scan series (first time / follow-up)	
Total ALP first visit	
Total ALP after 3 months	
Lumbosacral spine X-ray (osteoblastic / no lesions / equivocal)	
LFT - AST	
LFT - ALT	
Renal - Na+	
Renal - K+	
Renal - urea	
Renal - creatinine	
Renal - eGFR	
NHIS/private insurance (Y/N)	
Pathology laboratory for biopsy report	
